# A Case of Lichen Planus Masquerading as Guttate Psoriasis

**DOI:** 10.7759/cureus.11206

**Published:** 2020-10-27

**Authors:** Abraar Muneem, Surav M Sakya, Usman Asad

**Affiliations:** 1 Medicine, Penn State College of Medicine, Hershey, USA; 2 Dermatology, St. Anthony Hospital, Oklahoma City, USA

**Keywords:** lichen planus, eczema, guttate psoriasis, misdiagnosis, steroid therapy, family medicine, dermatology

## Abstract

Lichen planus (LP) is a chronic inflammatory disease that affects the skin, hair, nails, and mucous membranes, with variants such as drug-induced lichen planus, which is triggered by medications such as angiotensin-converting enzyme (ACE) inhibitors and antimalarials. Guttate psoriasis (GP), a clinical variant of psoriasis, is associated with streptococcal infections and presents with drop-like papules on the trunk and proximal extremities. In this report, we present a case of LP in an atypical location masquerading as GP and the importance of prompt dermatological referral to improve the patient’s quality of life. Coexistence and similarities between several variants of LP and plaque psoriasis have been seen in the literature. However, to our knowledge, our report is the first to show LP specifically mimicking GP.

## Introduction

Lichen planus (LP) is a chronic inflammatory disease that affects the skin, hair, nails, and mucous membranes. LP classically presents as pruritic, purple/violaceous, polygonal, flat-topped papules or plaques [1]. Drug-induced lichen planus (DILP), a clinical variant of LP, is generally triggered by hydrochlorothiazide, beta-blockers, angiotensin-converting enzyme (ACE) inhibitors, antimalarials, and NSAIDs and presents with more of a psoriasiform or eczematous appearance [1]. Guttate psoriasis (GP), a clinical variant of psoriasis, presents with drop-like papules; it is thought to be associated with human leukocyte antigen (HLA) genetic mutations and Koebnerization [2]. Notable risk factors are group A streptococcal and upper respiratory infections and medications, such as beta-blockers, tumour necrosis factor alpha (TNF-a) inhibitors, and antimalarials [2]. GP generally occurs in a symmetric distribution, affecting primarily the trunk and proximal extremities. In this report, we present a case of lichen planus masquerading as guttate psoriasis and the importance of prompt dermatological referral to improve the patients’ quality of life.

## Case presentation

A 76-year-old Caucasian woman with a past medical history of type 2 diabetes mellitus, hyperlipidemia, right iliac artery occlusion, COPD, class 1 obesity (BMI= 32.1) and squamous cell carcinoma of the lung status post chemotherapy in 2012 presented to our family medicine clinic with a 10-day history of an itchy rash on her lower extremities. She denied any fever, chills, sore throat or upper respiratory infections. Her daily medications were amlodipine, atorvastatin, lisinopril, metformin, and pantoprazole. She was allergic to aspirin, codeine, and gadolinium-based contrast media. She denied any recent sick contacts, traveling, or new use of any medications, detergents, soaps or lotions. Family history was unremarkable for dermatological or autoimmune conditions. Physical exam revealed multiple 3-8 mm erythematous papules with scaling, flaking, and scabbing present on the bilateral lower extremities. Uncertain of the etiology, the clinic prescribed our patient 0.1% triamcinolone cream to be applied twice daily.

Three weeks later, the patient’s rash and pruritus had worsened. Violaceous papules with excoriations and erythematous macules with no apparent distribution were seen on the lower extremities. The papules were blanchable but neither tender nor warm to touch. A clinical diagnosis of guttate psoriasis was made. The patient was prescribed a seven-day course of 0.05% betamethasone cream for daily use and was referred to dermatology. 

**Figure 1:**
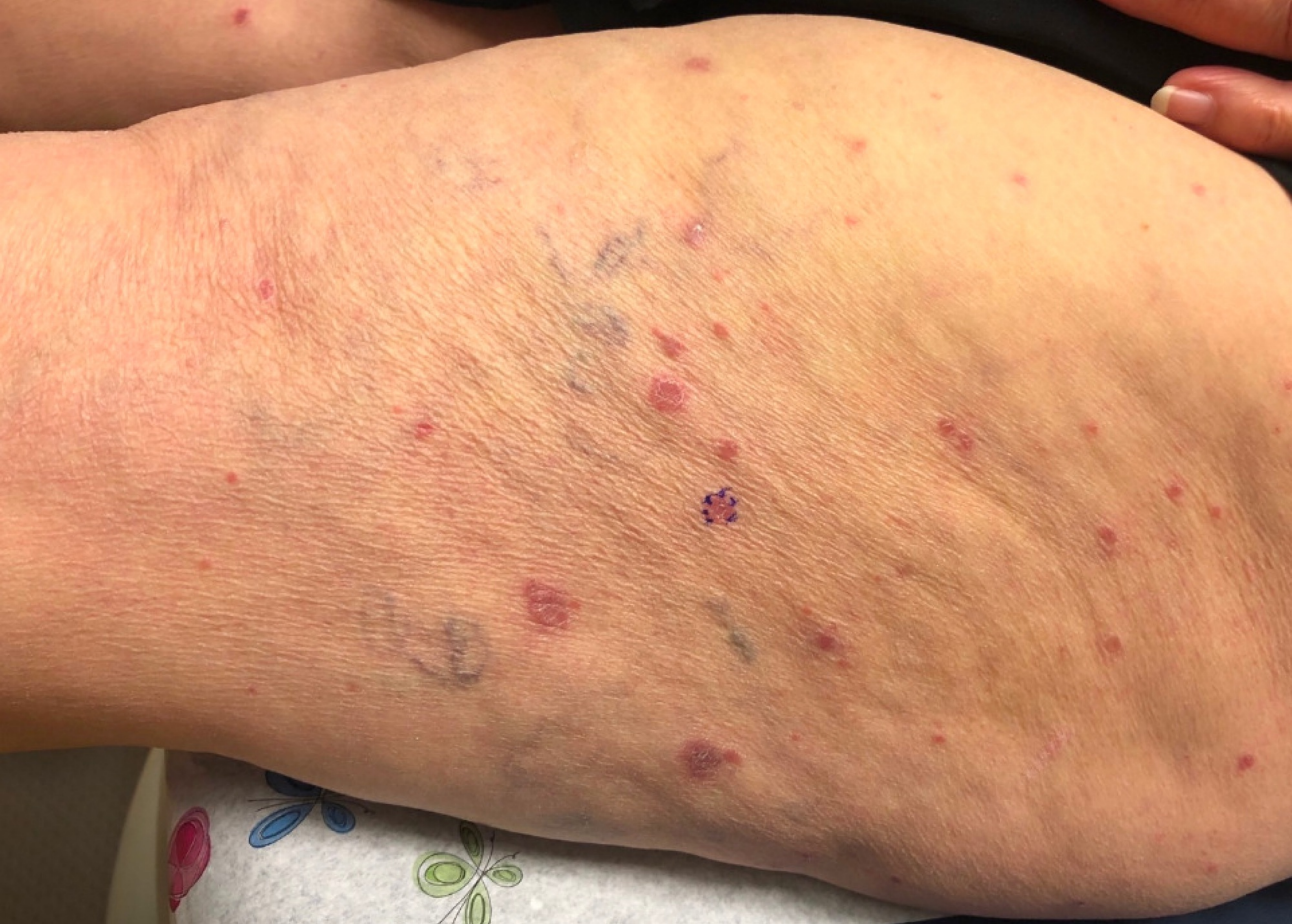
A 76-year-old female patient with pink papules with collarette of scale on the lateral thighs and legs bilaterally.

At her follow-up dermatology visit two weeks later, she reported only mild improvement of her pruritus. Physical exam showed multiple pink papules with collarette of scale on the lateral thighs and legs bilaterally (Figure 1).

The dermatologists classified it as a lichenoid dermatitis, possibly lichen planus or lichenoid drug eruption. The patient was instructed to increase the use of the betamethasone cream to twice daily until her shave biopsy results. The shave biopsy revealed an interface dermatitis with numerous colloid bodies, wedge-shaped hypergranulosis, overlying hyperkeratosis and a few eosinophils within the dermis. The patient was informed of the diagnosis of lichen planus with possible drug eruption and was recommended to continue the betamethasone cream twice daily until resolution of her rash. At her follow-up visit three months later, she reported complete resolution of her rash and pruritus after one month of betamethasone use. 

## Discussion

Our patient was initially diagnosed with GP by the family medicine clinic. A clinical variant of plaque psoriasis, it is often seen in children and adolescents and presents with drop-like lesions measuring 2-6 mm usually on the trunk or proximal extremities. Histopathology shows thick, mounded parakeratosis adherent to the epidermis with neutrophilic microabscesses. In contrast, LP shows orthohyperkeratosis, wedge-shaped granulosis, irregular acanthosis with saw-toothed rete ridges, and apoptotic keratinocytes confined to the basal layer. LP is most commonly seen on the oral mucosa (75%) and the ventral aspect of the wrist and forearms and is distinctly apparent as pruritic, purple, polygonal, flat-topped papules or plaques. DILP is distinguished by the presence of parakeratosis and eosinophils and clinically presents with a more eczematous or psoriasiform nature. Our patient’s biopsy ruled out GP and was more consistent with LP. While DILP was considered due to the presence of dermal eosinophils, our patient had not started any new medications recently. She had been using her ACE inhibitor for one year with no complications. While not common, LP has been shown in the literature to be present on the lower extremities; our patient’s case was atypical in that regard, but not unheard of [3-6].

Coexistence and similarities between several variants of LP and plaque psoriasis have been seen in the literature. However, to our knowledge, our report is the first to show LP specifically mimicking GP. Delaney and Smith reported a case of LP coexisting with plaque psoriasis in an African American patient; they believe coexistence of these two conditions may be underreported in the literature due to the misdiagnosis of LP as atypical plaques of psoriasis, particularly in skin of color [7]. In a survey of 27 participating Italian dermatological services, Naldi et al. collected 711 cases of LP, of which 12 cases had associated psoriasis [8]. Shiohara et al. reported a case of a patient who developed lesions consistent with LP with spontaneous improvement of preexisting psoriatic lesions; later, the patient experienced exacerbation of his preexisting psoriasis with the disappearance of LP. The authors concluded that although LP and psoriasis were seen to coexist in their patient, their clinical course may not be independent of each other, but rather could be affected by common immunological factors such as interferon gamma (IFN-y) [9]. Hypertrophic LP has also been confused with psoriasis and usually does not respond to therapy with biologics, highlighting the importance of histology and biopsies [10]. Linear LP has been shown to coexist with psoriasis as well; in one case, CD8+ T lymphocytes predominated over CD4+ T lymphocytes in the dermal infiltrates of the linear LP lesion, while in the psoriatic lesion, the infiltrates were mainly composed of CD4+ T lymphocytes. Again, it was speculated that some kind of antigenic stimuli may have triggered interferon-alpha (IFN-a) overproduction to trigger coexistence of these conditions [11]. A relationship may exist between the two diseases, either predisposing a patient with one to the other, or making one more resistant to the other [12]. Treatments for lichen planopilaris (such as hydroxychloroquine), seen in association with LP, may trigger psoriasis. Conversely, some treatments for psoriasis, such as antitumor necrosis factor inhibitors, have been reported to induce lichen planopilaris. One case reported successful treatment of existing lichen planopilaris and psoriasis with brodalumab, an interleukin-17 receptor (IL-17R) antagonist [13].

## Conclusions

Our patient’s symptoms only improved after the dermatology clinic increased her treatment regimen to twice-daily use of the betamethasone. Our patient experienced distress from the reaction and had to deal with the pruritus for several more weeks after leaving the family medicine clinic; a total of 50 days passed before a diagnosis was confirmed to the patient. An earlier referral and biopsy may have shortened the process and, in future circumstances, could prevent instances of inappropriate treatment. When uncertain of the etiology, prompt referral to a dermatologist is highly recommended to maximize the individual’s medical and psychological well-being, as treatments and disease courses can vary greatly even between similar-looking cutaneous disorders.
